# Effects of macro- and micro-nutrients on momentary and season-long feeding responses by select species of ants

**DOI:** 10.1038/s41598-024-56133-y

**Published:** 2024-03-08

**Authors:** Asim Renyard, Claire Gooding, Jaime M. Chalissery, Jonathan Petrov, Gerhard Gries

**Affiliations:** Department of Biological Sciences, Simon Fraser University, 8888 University Drive, Burnaby, BC V5A 1S6 Canada

**Keywords:** Hymenoptera, Formicidae, Ants, Foraging, Nutrient consumption, Diet choice, Animal behaviour, Behavioural ecology

## Abstract

Few studies have investigated the relative contribution of specific nutrients to momentary and season-long foraging responses by ants. Using western carpenter ants, *Camponotus modoc,* and European fire ants, *Myrmica rubra,* as model species, we: (1) tested preferential consumption of various macro- and micro-nutrients; (2) compared consumption of preferred macro-nutrients; (3) investigated seasonal shifts (late May to mid-September) in nutrient preferences; and (4) tested whether nutrient preferences of *C. modoc* and *M. rubra* pertain to black garden ants, *Lasius niger*, and thatching ants, *Formica aserva*. In laboratory and field experiments, we measured nutrient consumption by weighing Eppendorf tubes containing aqueous nutrient solutions before and after feeding by ants. Laboratory colonies of *C. modoc* favored nitrogenous urea and essential amino acids (EAAs), whereas *M. rubra* colonies favored sucrose. Field colonies of *C. modoc* and *M. rubra* preferentially consumed EAAs and sucrose, respectively, with no sustained shift in preferred macro-nutrient over the course of the foraging season. The presence of a less preferred macro-nutrient in a nutrient blend did not diminish the blend’s ‘appeal’ to foraging ants. Sucrose and EAAs singly and in combination were equally consumed by *L. niger,* whereas *F. aserva* preferred EAAs. Baits containing both sucrose and EAAs were consistently consumed by the ants studied in this project and should be considered for pest ant control.

## Introduction

Adequate nutrition is vital for colony fitness in ants^1,2^, affecting colony functioning, brood production and development, and worker survival^2^. The availability of carbohydrates as an energy source affects both the activity^3–6^ and the longevity of worker ants^3,4,6–10^, which, in turn, modulate foraging activities and aggressiveness of ant colonies^3–6,11,12^. Protein sources, in combination with carbohydrates, are essential for egg production by queens and brood development^3,4,13–16^, but in high amounts are toxic to workers^7–10^.

Ant colonies face challenges to meet their nutritional needs. Foragers must locate and recognize required nutrient resources, and integrate their own nutritional needs with those of their nestmates^2^. Ants locate food resources by responding to their odor plumes^17–24^, or by following trail pheromone deposited by forager ants^25^. The pattern of nutrient collection by ants may shift over time, with more carbohydrate- or protein-rich resources collected at different times of year^26–29^. Shifts in nutrient preference may be caused by demographic shifts in ant colonies or shifts in nutrient availability in the ants’ habitat. Experimentally increased amounts of colony brood mobilized foraging in *L. niger* workers^30^, prompted more food and more protein collection by *Rhytidoponera* ants^31,32^*,* and more protein consumption by *Ochetellus glaber* ants^33^. Ant colonies that have plenty of readily available nutrients preferentially seek foods containing scarcer nutrients^34,35^.

Ants assess food quality through the presence and concentration of certain macro-nutrients (proteins, carbohydrates, lipids) and micro-nutrients (e.g., salts, vitamins). In aphid honeydew, ants recognize the different types of sugar molecules^36–41^ (e.g., sucrose, melezitose) and generally they prefer resources with high sugar concentration^39,41,42^. Ants also require proteins, and obtain amino acids—the ‘building blocks’ of proteins—by e.g. (*i*) ingesting free amino acids from plant nectar^43–46^, (*ii*) digesting proteins from insect prey^47^, and (*iii*) by acquiring the amino acids that symbiotic gut microbes produce from nitrogenous waste^15,48^. Ants prefer essential to non-essential amino acids^49^, and recognize when specific amino acids are lacking^49^. Ants also feed on certain oils^38^, and recognize distinct fatty acids (e.g., oleic acid) and glycerides (e.g., 1,2-diolein) that are present on the surface of insect prey, deceased insects, and seed elaiosomes, and that serve as pick-up cues by ants^50–53^. Furthermore, ants recognize and consume micro-nutrients, including salts^54–57^ (e.g., NaCl) and some B-vitamins^58^.

Very few studies have comprehensively examined the ants’ preferences for specific macro- and micro-nutrients or for these nutrients as components of complex nutrient blends^37,49,58^. Although seasonal shifts in nutrient preference by ants have been demonstrated^26–29^, these nutrients were often presented as complex blends (e.g., tuna, fruit conserves) with sometimes different physical properties, making it difficult to attribute the ants’ preferential feeding responses to any one nutrient component.

Previously, we have determined nutrient preferences of two generalist ant species, the western carpenter ant, *Camponotus modoc*, and the European fire ant, *Myrmica rubra*^41^. *Camponotus modoc* inhabits temperate forests along the western coast of North America^59^ and excavates nests in the wood of conifer trees^60^. *Myrmica rubra* is an aggressive soil-dwelling ant that is native to Eurasia but has invaded the east and west coasts of North America^61^. While both species prefer sucrose to other saccharides^41^, and both species prefer essential to non-essential amino acids (AR, unpublished data), only *C. modoc* also consumes urea (AR, unpublished data). *Lasius niger* is a widespread^62,63^, temperate, soil-dwelling ant that regularly tends aphids and prefers aphid-derived sugars such as melezitose to common sugars such as sucrose^36,64^. *Lasius niger* also preferentially feeds on diverse amino acid blends^64,65^ but any potential preference for specific amino acids is not known. *Formica aserva* is a brood-raiding ant, nesting in woody debris^66^ such as stumps^67^. It tends aphids^68^ and collects insect prey^69^ but nutrient preferences are not yet documented. We selected these four species to represent ants in diverse taxa with contrasting morphology and body size, life history traits, and habitat preferences.

Here, we investigated momentary (ad hoc), and seasonal, nutrient preferences of *C. modoc* and *M. rubra* colonies*,* and determined whether their observed nutrient preferences apply to other ant taxa. Specifically, we: (1) tested nutrient consumption within groups of either macro-nutrients or micro-nutrients; (2) compared consumption of preferred macro-nutrients; (3) investigated potential seasonal shifts in nutrient preferences; and (4) tested whether nutrient preferences of *C. modoc* and *M. rubra* pertain to *L. niger* and *F. aserva.*

## Materials and methods

### Maintenance of laboratory ant colonies

We collected and maintained nine colonies of *C. modoc* between 2016 and 2020, as reported^70^. Briefly, we removed infested log sections from coniferous forests near Squamish (British Columbia), and transferred these sections to large plastic bins (64 × 79 × 117 cm) kept in an outdoor, under-cover area of the Science Research Annex at Simon Fraser University. All colonies experienced natural weather and light cycles which can be important for colony survival^71^. Bins were connected to glass containers (30.5 × 26 × 50.8 cm) which served as the ants’ foraging area. The upper inner bin and container walls were coated with an equal mix of Vaseline (Unilever, London, UK) and paraffin oil (Anachemia, Lachine, QC H8R1A3, CA) to prevent ant escape. Ants were provisioned with apples, deceased cockroaches, and 20% sugar water ad libitum. Containers and bins had mesh covered holes to allow air exchange.

We collected and reared invasive *M. rubra* similar to previous reports^41,72^, with some modifications. In the summer of 2021, 10 colonies were dug up with their nesting soil at Inter-river Park (North Vancouver, BC, CA), and temporarily placed in glass jars (1L). Colonies were then transferred to separate glass containers (26 × 21 × 40.6 cm), with mesh-covered holes in container lids, and upper inner container walls coated with Vaseline and paraffin oil. Colonies were maintained indoors at 25–30 °C under a natural daylength cycle, and were provisioned with food as described above. The soil surface of containers served as the ants’ foraging area. Every two weeks, water was added to the soil to ensure adequate moisture content. Colonies were kept indoors, instead of outdoors, to minimize the risk of ant escape on university campus.

### Preparation of test stimuli

Prior to experiments, test stimuli were prepared by weighing nutrients (TR 204 scale; Denver Instrument Company, CO, USA; see Table 1 for number of test stimuli, and Table S1 for nutrient compositions) and mixing them into water, accounting for ~ 50% of the final volume. Once nutrients were dissolved, distilled water was added until the desired weight by volume solution (w/v) was reached. Aliquots (1 mL) of solutions were pipetted into 1.5-mL Eppendorf tubes (Thermo Fisher Scientific, Waltham, MA 02451, USA) and kept frozen until use in bioassays. For each experiment, we prepared as many tubes, including evaporation control tubes (see below), as required for testing stimuli consumption by all colonies. Sucrose and essential amino acids (EAAs) were selected as nutrients based on previous studies^41^, and EAAs were assembled drawing on both an article^15^ and personal communication with its senior author. Fatty acids (oleic, linoleic, linolenic) and glycerides (1,2 diolein, triolein) were tested because they serve as pickup cues for ants^50,52,73^. Selections of sterols, and their approximate ratio, were based on reported dietary needs of insects^47^. Salts and vitamins were tested at equal ratio drawing on the composition of a synthetic ant diet^74^ (see Table S1 for compositions of test stimuli).

**Table 1:** Research objectives (O), stimuli tested, numbers of replicates (n) run, and ant species bioassayed in experiments 1–24.

Exp. #	Stimuli^a^ tested	n (species tested)
(O1) Assess consumption of various macro- and micro-nutrients		
1–2	Water *vs* 0.625%^b^ *vs* 1.25% *vs* 2.5% sucrose	9 (*C. modoc*); 10 (*M. rubra*)
3–4	Water *vs* Tween 80 (0.05%) *vs* 0.5% glycerides *vs* 1.0% glycerides	9 (*C. modoc*); 9 (*M. rubra*)
5–6	Water *vs* Tween 80 (0.05%) *vs* 1.25% fatty acids *vs* 2.5% fatty acids	7 (*C. modoc*); 9 (*M. rubra*)
7–8	Water *vs* Tween 80 (0.05%) *vs* 0.5% sterols *vs* 1.0% sterols	7 (*C. modoc*); 9 (*M. rubra*)
9–10	Water *vs* 0.25% *vs* 0.5% *vs* 1.0% salts	8 (*C. modoc*); 9 (*M. rubra*)
11–12	Water *vs* 0.25% *vs* 0.5% *vs* 1.0% vitamins	8 (*C. modoc*); 10 (*M. rubra*)
(O2) Compare consumption between preferred macronutrients		
13 &15	Water *vs* urea (2.5%) *vs* EAAs (0.55%) *vs* sucrose (2.5%) *vs* blend (5.55%); unadjusted^c^	8 (*C. modoc*); 9 (*M. rubra*)
14 & 16	Water *vs* urea (5.55%) *vs* EAAs (5.55%) *vs* sucrose (5.55%) *vs* blend (5.55%); adjusted^c^	9 (*C. modoc*); 8 (*M. rubra*)
17 & 19	Water *vs* urea + EAAs (2.5%; 0.55%) *vs* urea + sucrose (2.5%; 2.5%) *vs* EAAs + sucrose (0.55; 2.5%) *vs* blend (5.55%); unadjusted	9 (*C. modoc*); 9 (*M. rubra*)
18 & 20	Water *vs* urea + EAAs (4.55%; 1.0%) *vs* urea + sucrose (2.775%; 2.775%) *vs* EAAs (1.0%) + sucrose (4.55%) *vs* blend (5.55%); adjusted	9 (*C. modoc*); 9 (*M. rubra*)
(O3) Evaluate seasonal nutrient preferences		
21–22	Water *vs* EAAs (5.55%) *vs* sucrose (5.55%) vs blend (5.55%)	12–13 (*C. modoc*); 10 (*M. rubra*)
(O4) Investigate nutrient preferences of other ant taxa		
23–24	Water *vs* EAAs (5.55%) *vs* sucrose (5.55%) *vs* blend (5.55%)	10 (*L. niger*); 10 (*F. aserva*)

^a^For detailed stimulus compositions and suppliers of nutrients see Table S1.

^b^Percentages are expressed as weight by volume (w/v).

^c^‘Unadjusted’ and ‘adjusted’ refer to solutions containing one or two types of macro-nutrients, each type with the same percentage concentration as in the ternary blend (‘unadjusted blend’), or with its concentration increased to match the total concentration of the ternary blend (‘adjusted blend’).

### General protocol for laboratory bioassays

**Figure 1:**
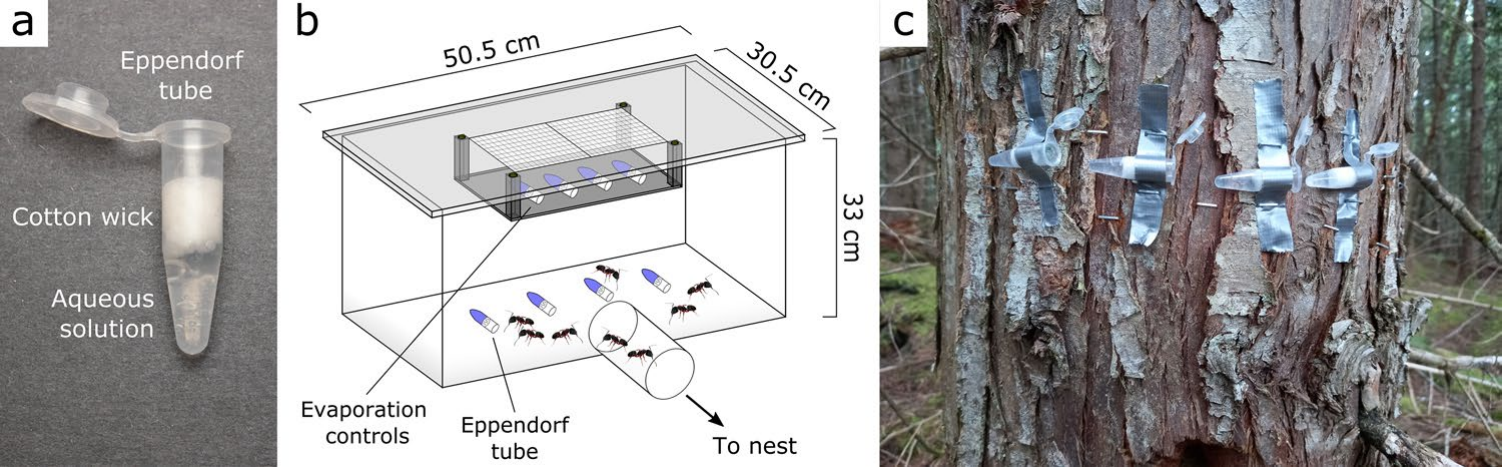
Design of laboratory and field experiments for testing comparative food consumption by ants. (**a**) Photograph of an Eppendorf tube containing an aqueous nutrient solution retained by a piece of dental cotton wick, enabling ants to consume the liquid bait without spillage. (**b**) Bioassay container for carpenter ants, *Camponotus modoc,* fitted with 1.5-mL Eppendorf tubes containing aqueous nutrient solutions. Similar methodology was used for testing food consumption by European fire ants, *Myrmica rubra*^41^. Evaporation control tubes were placed on a small platform suspended from the container ceiling. (**c**) Eppendorf tubes affixed to a tree to test for preferential food consumption by *C. modoc* and black garden ants, *Lasius niger.* For field experiments with *M. rubra* and *Formica aserva*, Eppendorf tubes were placed on the ground.

On any experimental day, test stimuli were removed from the freezer, thawed, and vortexed, thus ensuring that all solutes were dissolved. Eppendorf tubes were then stuffed with a 1-cm-long piece of a cotton dental wick (Richmond Dental & Medical, Charlotte, NC 28205, USA) to allow nutrient consumption by ants without spillage (Fig. 1a). For each test stimulus, two tubes were prepared: one for ant consumption and another for tracking passive water evaporation during bioassays. All tubes were weighed just prior to, and at the end of, bioassays. In each bioassay replicate, we tested the consumption response of a different colony and prepared as many Eppendorf tubes as stimuli were tested (typically 4–5), with each tube containing a specific nutrient solution or a plain water control. Prior to the onset of a bioassay, tubes were weighed and uncapped, and then presented to the ants, allowing them to forage for 4–6 h (see below). Bioassay times for *C. modoc* and *M. rubra* colonies were set to 4 h and 6 h, respectively, accounting for differences in worker size^60,61^ and considering the time that was needed to obtain measurable consumption rates in preliminary experiments. Colonies for laboratory experiments were randomly selected on each experimental day, and were given at least 36 h between experiments. Each colony experienced a particular set of test stimuli only once. All laboratory experiments were conducted between June and early September.

Bioassays with *C. modoc* (and *M. rubra* below) were run mostly on warm and sunny days when colonies are most active (AR, pers. obs.). Prior to bioassays, colonies were deprived of cockroaches and apples for 24 h, and of sugar water for 4 h (the maximum time elapsed before ants attempted to chew out of their containers). Bioassays were run in plexiglass containers (50.5 × 30.5 × 33 cm) covered by lids with mesh holes to allow ventilation (Fig. 1b). Tubes were taped, with positions randomly assigned and spaced equidistantly in an arc, to the container bottom 22 cm away from the container entrance hole. Corresponding evaporation control tubes were taped to a plexiglass platform suspended from the container lid. Just prior to initiating a bioassay, all tubes were uncapped and each container was connected via Tygon® tubing (diam.: 2.54 cm) and barbed plumbing connectors (diam.: 2.54 cm) to a *C. modoc* housing bin, allowing ants to freely forage in a container. Bioassay replicates were run for 4 h but were terminated sooner if ants had completely consumed the test solution of any one tube. Bioassay containers were cleaned with hexane and ethanol (70%), and plumbing fixtures and Tygon® tubings were washed with soapy water.

For bioassays with *M. rubra,* nest containers were co-opted as bioassay containers, and colonies were food-deprived for 24 h to motivate foraging. For each replicate, Eppendorf tubes were taped, randomly assigned, to the edge of a jar lid (diam: 15 cm), and corresponding evaporation control tubes were taped, inaccessible to ants, to the underside of container lids. Bioassay replicates were initiated by uncapping all Eppendorf tubes, and placing jar lids with Eppendorf tubes on the soil surface inside bioassay containers. Bioassays were run for 6 h but were terminated sooner if ants had consumed the entire test solution in an Eppendorf tube. Between replicates, jar lids were washed with soapy water.

### Protocol of field experiment

In preparation for field experiments, Eppendorf tubes with nutrient solutions for ant consumption, and evaporation control tubes, were thawed, weighed, and then transported to the field in a cooler. Tubes were spaced around the entrance of ant nests or next to ant foraging trails, with tube positions randomly assigned in each replicate. For *C. modoc,* Eppendorf tubes were affixed 5 cm apart to trees or logs housing a *C. modoc* nest (Fig. 1c). For *L. niger,* we located nests at the base of trees, and affixed tubes next to each other on the trunk of trees alongside the ants’ foraging trails. For *M. rubra,* tubes were placed 5 cm apart around the entrance of subterranean nests. For *F. aserva*, tubes were placed on top of tree stumps that contained an ant nest. For all field studies, evaporation control tubes were placed in Tupperware containers (15 × 9 × 10 cm) with a mesh-covered hole in the lid, and containers were set near ant nests. Replicates with colonies of *M. rubra* (n = 10; repeated on 7 dates)*, L. niger* (n = 10), *C. modoc* (n = 13; repeated on 6 dates), and *F. aserva* (n = 10) were run for 4 h, 16 h, 24 h, and 24 h, respectively. Bioassays were run between 10:00–14:00 for *M. rubra,* 17:00–09:00 for *L. niger*, and between 11:00–11:00 for both *C. modoc* and *F. aserva.* Experimental time periods for each species were set according to time periods needed in preliminary studies to obtain measurable consumption rates. After replicates were terminated, tubes were capped, transported to the laboratory in a cooler, and weighed. All 4- to 24-h field studies were run on warm and sunny days with observable ant activity. Experiments with *C. modoc* and *M. rubra* were run from late May to mid-September, and experiments with *L. niger* and *F. aserva* were run in June and August, respectively.

### Specific experiments

#### Assessing consumption of various macro- and micro-nutrients (Exps. 1–12; Lab)

In experiments 1–2 (Table 1), we offered colonies of *C. modoc* and *M. rubra* a choice between aqueous sucrose solutions at three concentrations (0.625%, 1.25%, and 2.5% w/v), and a water control.

Experiments 3–8 (Table 1) tested consumption of lipid-related nutrients by *C. modoc* and *M. rubra* colonies. Each of three lipid types (glycerides, fatty acids, sterols) consisted of 2–4 constituents (Table S1) which were formulated in an aqueous solution at two concentrations (Exps. 3–4: glycerides: 0.5%, 1.0% w/v; Exps. 5–6: fatty acids: 1.25%, 2.5% w/v; Exps. 7–8: sterols: 0.5%, 1.0% w/v), using Tween 80 as the emulsifier. In each experiment, both Tween 80 in water, and water, served as control stimuli.

Experiment 9–12 (Table 1) tested consumption of micro-nutrient salts or vitamins by *C. modoc* and *M. rubra* colonies. Each type of micro-nutrient consisted of 7–11 constituents (Table S1) which were dissolved in water at three concentrations (Exps. 9–10: salts: 0.25%, 0.5%, 1.0% w/v; Exps. 11–12: vitamins: 0.25%, 0.5%, 1.0% w/v), with water serving as the control stimulus in each experiment.

#### Comparing consumption of preferred macro-nutrients (Exps. 13–20; Lab)

Experiments 13–20 (Table 1) compared consumption of macro-nutrients that *C. modoc* or *M. rubra* colonies were previously shown to preferentially consume, including urea (AR, unpublished data), essential amino acids (EAAs; AR, unpublished data), and sucrose^41^. In experiments 13–16, aqueous solutions of urea, EAAs, and sucrose were tested singly and in ternary combination, with plain water as the control stimulus. Single components were tested at the same ‘unadjusted’ concentration as in the ternary blend (urea 2.5%; EAAs 0.55%; sucrose 2.5% w/v) or at an ‘adjusted’ concentration (5.55% w/v) that equalled the total concentration of the ternary blend (5.55%). Each component in the ternary blend was tested at the lowest concentration found effective in pre-screening experiments (see Result of Exps. 1–12; AR, unpublished data). In experiments 17–20 (Table 1), aqueous solutions of urea, EAAs, and sucrose were tested in all binary and ternary combinations, again with plain water as the control stimulus. Binary combinations were tested at the same ‘unadjusted’ concentration as in the ternary blend (urea [2.5%] + EAA [0.55%]; urea [2.5%] + sucrose [2.5%]; EAA [0.55%] + sucrose [2.5%]) or at an ‘adjusted’ concentration (urea [4.55%] + EAA [1.0%]; urea [2.775%] + sucrose [2.775%]; EAA [1.0%] + sucrose [4.55%]) that equalled the total concentration of the ternary blend (5.55%).

#### Evaluating potential seasonal shifts in nutrient consumption (Exps. 21–22; Field)

Experiments 21**–**22 (Table 1) investigated potential seasonal shifts in nutrient preferences exhibited by field colonies of ants. We worked with 13 colonies of *C. modoc* and 10 colonies of *M. rubra* located along the Mamquam forest service road (near Squamish, BC, Canada) and at Inter River Park (District of North Vancouver, BC, Canada), respectively. Drawing on results of preceding experiments that both *C. modoc* and *M. rubra* had preferentially consumed the ‘adjusted’ binary blend of EAA + sucrose (see “Results”), we offered each ant colony four Eppendorf tubes that contained: (1) EAA (5.55%); (2) sucrose (5.55%), (3) EAA (1.0%) + sucrose (4.55%); and (4) plain water (control). Throughout the summer season, we measured nutrient consumption by colonies in circa 3-week intervals on six dates for *C. modoc* colonies (18 June 2021 to 07 September 2021), and on seven dates for *M. rubra* colonies (21 May 2021 to 13 September 2021).

#### Investigating nutrient consumption of *L. niger* and *F. aserva* (Exps. 23–24; Field)

We worked with 10 field colonies each of *L. niger* and *F. aserva* that were located on the Burnaby campus of Simon Fraser University and along the Mamquam forest service road (see above), respectively. Each nest was offered four Eppendorf tubes that contained: (1) EAAs (5.55%); (2) sucrose (5.55%), (3) EAAs (1.0%) + sucrose (4.55%); and (4) plain water (control).

### Statistical analyses

To calculate the amount of each nutrient solution that was consumed by a colony, we first determined the weight loss of the corresponding evaporation control solution, and then subtracted this value from the weight loss of the test solution. To account for differences in colony size and foraging activity between colonies, we analysed proportions, rather than absolute amounts, of nutrient solutions consumed. To obtain proportional consumption data for a colony in any experimental replicate, we divided the amount (weight) of each nutrient solution consumed by the total amount of all nutrient solutions consumed. When there had been little feeding activity by a colony, some consumption data became less than zero (~ −7 mg) following weight loss subtraction due to water evaporation measured in evaporation controls (see above). As evaporation control tubes were close to the arena vent (Fig. 1b), these small negative values could be due to slightly elevated rates of evaporation. As there could not be ‘negative feeding’ on a nutrient solution by a colony, we considered these less-than-zero values to be zero. We included replicates in data analyses when a colony had positive consumption responses for at least two of all the nutrient solutions that were tested in that replicate. Using a beta distributed generalized linear mixed model (GLMM), we applied a standard transformation to restrict our data between the bounded interval of 0 and 1. In experiments 1–20 and 23–24, we fit proportion consumed as our response variable and treatment as our predictor, with ant colony as a random intercept. For experiments 21–22, we fit proportion consumed as our response variable and treatment, date, and interaction between treatment and date as predictors, with ant colony as a random intercept. We evaluated the significance of predictors, using likelihood ratio tests and made Tukey adjusted pairwise comparisons between mean proportional consumption values between treatments.

Data^75^ were analysed and graphed using R (v. 4.2.2) and R studio (v. 2022.07.1 + 554)^76^. Data were processed using functions from the tidyverse^77^ and the plyr package^78^. GLMMs were fit using the glmmTMB package^79^, and model fit, residual normality, variance, and over/under dispersion patterns were inspected, using the DHARMa package^80^. We obtained estimated marginal means and 95% confidence intervals using the emmeans package^81^. We produced graphics using the ggplot2 package^82^ and completed figure assemblies in Inkscape (v. 1.0.2).

## Results

### Assessing consumption of various macro- and micro-nutrients (Exps. 1–12; Lab)

Concentrations of sucrose in aqueous solutions affected their consumption by *C. modoc* colonies (χ^2^ = 8.75, d.f. = 3, p = 0.03; Fig. S1a) and by *M. rubra* colonies (χ^2^ = 54.18, d.f. = 3, p < 0.0001; Fig. S1b). Colonies of *C. modoc* preferentially consumed the 1.5% (w/v) sucrose solution, which was the only solution consumed significantly more than water. Colonies of *M. rubra* consumed significantly more of the 2.5% (w/v) sucrose solution than of any other solution including the water control (Table S2).

Glycerides in emulsified aqueous solutions did not prompt consumption by *C. modoc* colonies (χ^2^ = 2.03, d.f. = 3, p = 0.57; Fig. S2a) and *M. rubra* colonies (χ^2^ = 0.72, d.f. = 3, p = 0.87; Fig. S2b, Table S3).

Fatty acids in emulsified aqueous solutions significantly affected feeding responses of *C. modoc* colonies (χ^2^ = 8.46, d.f. = 3, p = 0.04; Figure S2c) and *M. rubra* colonies (χ^2^ = 8.90, d.f. = 3, p = 0.03; Fig. S2d). Increasing concentrations of fatty acids lowered consumption, with the 2.5% solution being consumed the least (Table S3).

Sterols in aqueous solutions did not affect consumption by *C. modoc* colonies (χ^2^ = 2.47, d.f. = 3, p = 0.48; Fig. S2e) but did affect consumption by *M. rubra* colonies (χ^2^ = 9.90, d.f. = 3, p = 0.02; Fig. S2f). Colonies of *M. rubra* consumed more of the 0.5% sterol solution than of the 1% sterol solution and the water control but not of the tween + water control (Table S3). The 1% sterol solution, Tween + water, and water all prompted comparable consumption (Table S3).

Salts in aqueous solutions did not affect consumption by *C. modoc* colonies (χ^2^ = 0.89, d.f. = 3, p = 0.83; Fig. S3a) but was a significant predictor of proportional consumption by *M. rubra* colonies (χ^2^ = 32.26, d.f. = 3, p < 0.0001; Fig. S3b). Increasing salt concentrations lowered consumption, with the 1% solution being consumed the least (Table S3).

Vitamins in aqueous solutions did not affect consumption by *C. modoc* colonies (χ^2^ = 0.10 d.f. = 3, p = 0.99; Fig. S3c) but affected consumption by *M. rubra* colonies (χ^2^ = 20.66, d.f. = 3, p < 0.001; Fig. S3d). Colonies of *M. rubra* equally consumed the 1% vitamin solution and plain water, both of which more than the 0.25% and 0.5% solution (Table S4).

### Comparing consumption of preferred macro-nutrients (Exps. 13–20; Lab)

**Figure 2:**
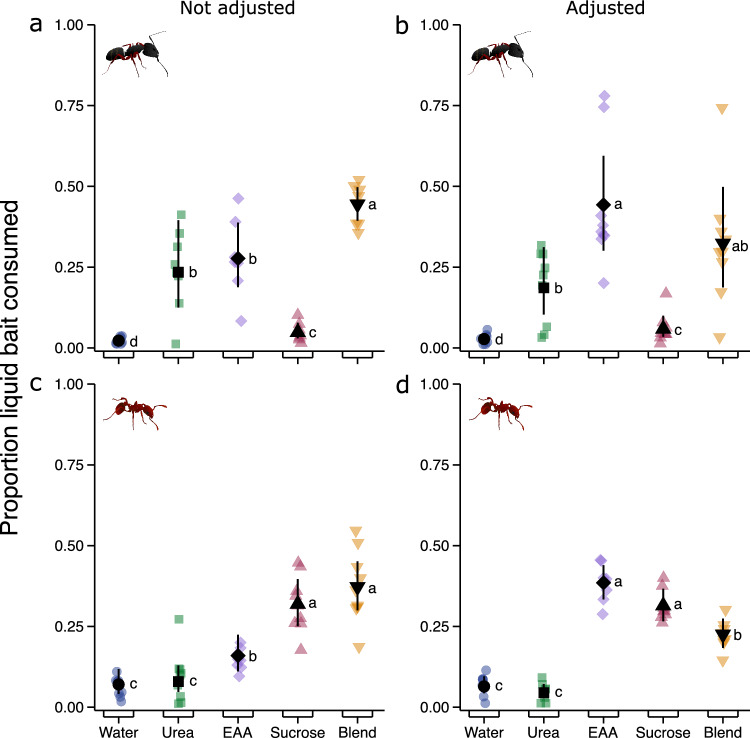
Comparative consumption of liquid food baits by colonies of *Camponotus modoc* carpenter ants [**a** (n = 8), **b** (n = 9)], and *Myrmica rubra* fire ants [**c** (n = 9), **d** (n = 8)]. Colonies were offered a choice of aqueous solutions of macro-nutrients—urea, essential amino acids (EAAs), and sucrose—that were presented singly and in a ternary blend (‘Blend’). The concentration of single macro-nutrients was either not adjusted [**a**, **c**; urea (2.5%); EAAs (0.55%); sucrose (2.5%) w/v] or adjusted (**b**, **d**; urea, EAAs, and sucrose all 5.55%) to the same total concentration as the ternary blend [urea (2.5%), EAAs (0.55%), and sucrose (2.5%) w/v]. Coloured symbols indicate consumption rates of individual colonies (replicates) and black symbols represent modelled estimated marginal means and 95% confidence intervals. Means with different letters are statistically different (p < 0.05) in pairwise comparisons (see Table S5).

Colonies of *C. modoc* and *M. rubra* differentially consumed 1- and 3-component aqueous solutions of urea, EAAs, and sucrose, and plain water (control stimulus) (Table 1) (*C. modoc:* unadjusted concentrations of nutrients in aqueous solutions: χ^2^ = 71.15, d.f. = 4, p < 0.0001, Fig. 2a; *C. modoc*: adjusted concentrations: χ^2^ = 57.72, d.f. = 4, p < 0.0001, Fig. 2b; *M. rubra*: unadjusted concentrations: χ^2^ = 61.59, d.f. = 4, p < 0.0001, Fig. 2c; *M. rubra*: adjusted concentrations: χ^2^ = 91.7, d.f. = 4, p < 0.0001, Fig. 2d). *Camponotus modoc* colonies preferentially consumed solutions containing urea, EAAs, or both (together with sucrose) (Fig. 2a,b). At adjusted nutrient concentrations, EAAs on their own and in ternary combination with urea and sucrose were most heavily consumed (Table S5). When nutrient concentrations were unadjusted, *M. rubra* colonies preferentially consumed sucrose, and sucrose in ternary combination with urea and EAAs. At adjusted nutrient concentrations, *M. rubra* colonies preferentially consumed single-nutrient solutions of EAAs and sucrose, followed by the ternary blend of EAAs, sucrose, and urea (Table S5).

**Figure 3:**
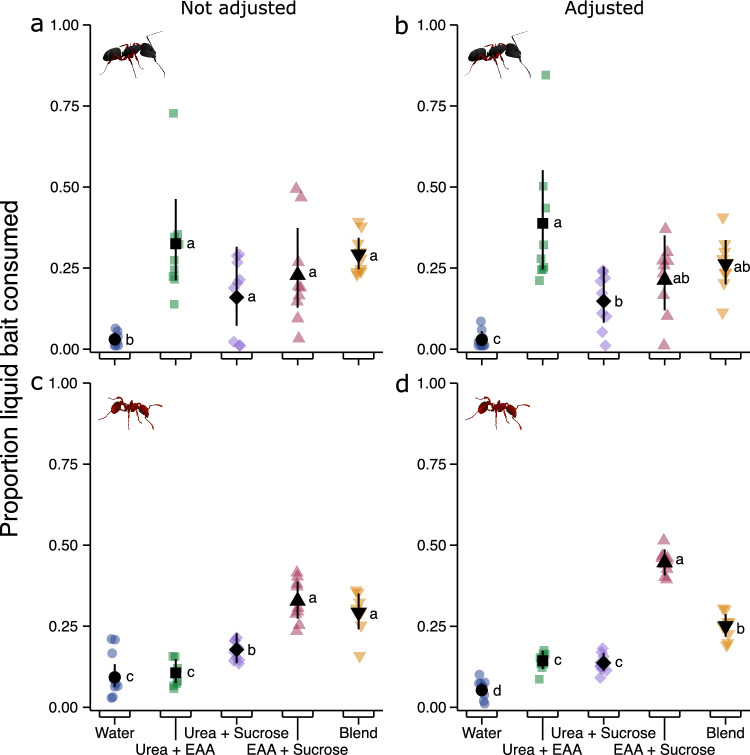
Comparative consumption of liquid food baits by colonies of *Camponotus modoc* carpenter ants [**a** (n = 9), **b** (n = 9)] and *Myrmica rubra* fire ants [**c** (n = 9), **d** (n = 9)]. Colonies were offered a choice of aqueous solutions of macro-nutrients—urea, essential amino acids (EAAs), and sucrose—that were tested in binary combinations and in a ternary blend (‘Blend’). The concentration of binary combinations was either not adjusted [**a**, **c**; urea (2.5%) and EAAs (0.55%); urea (2.5%) and sucrose (2.5%); EAAs (0.55%) and sucrose (2.5%)] or adjusted [**b**, **d**; urea (4.55%) and EAAs (1.0%); urea (2.775%) and sucrose (2.775%); EAAs (1.0%) and sucrose: 4.55%) to the same total concentration as the ternary blend [urea (2.5%), EAAs (0.55%), and sucrose (2.5%) w/v)]. Coloured symbols indicate responses of individual colonies (replicates) and black symbols represent modelled estimated marginal means and 95% confidence intervals. Means with different letters are statistically different (p < 0.05) in pairwise comparisons (see Table S6).

There was also differential consumption of macro-nutrients by *C. modoc* and *M. rubra* colonies when sucrose, EAAs, and urea were offered—at unadjusted and adjusted nutrient concentrations—in all possible binary and ternary combinations, along with plain water as the control stimulus (*C. modoc*: unadjusted concentrations: χ^2^ = 30.79, d.f. = 4, p < 0.0001, Fig. 3a; *C. modoc*: adjusted concentrations: χ^2^ = 30.71, d.f. = 4, p < 0.0001, Fig. 3b; *M. rubra*: unadjusted concentrations: χ^2^ = 61.329, d.f. = 4, p < 0.0001, Fig. 3c; *M. rubra*: adjusted concentrations: χ^2^ = 115.38, d.f. = 4, p < 0.0001, Fig. 3d). At unadjusted nutrient concentrations, *C. modoc* colonies equally consumed all binary and ternary nutrient blends, significantly preferring all of them to plain water (Fig. 3a; Table S6). At adjusted nutrient concentrations, *C. modoc* colonies consumed the blend of urea + EAAs significantly more than the blend of urea + sucrose, and water, but not significantly more than the blend of EAAs + sucrose, and the ternary blend (Fig. 3b; Table S6). At unadjusted nutrient concentrations, *M. rubra* colonies preferentially consumed the blend of EAAs + sucrose and the ternary blend, followed by blends of urea + sucrose and urea + EAAs, with the latter blend being consumed as little as water (Fig. 3c; Table S6). At adjusted nutrient concentrations, the blend of EAAs + sucrose was most heavily consumed, followed by the ternary blend, and by binary blends of urea + sucrose and urea + EAAs, which had similar levels of consumption, both significantly higher than water (Fig. 3d; Table S6).

### Evaluating potential seasonal shifts in nutrient consumption (Exps. 21–22; Field)

**Figure 4:**
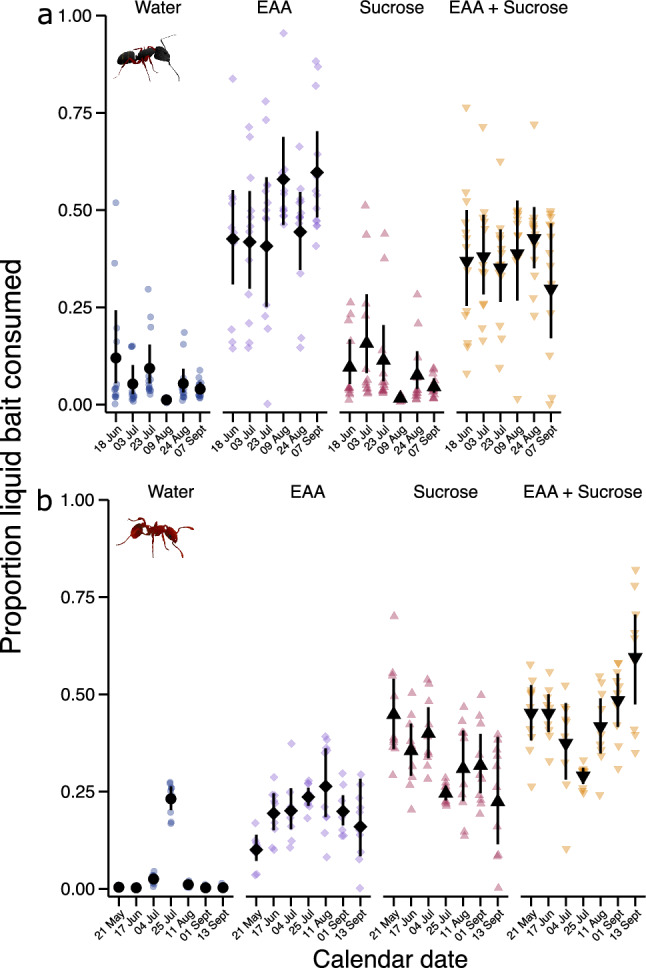
Comparative consumption of liquid food baits by field colonies of *Camponotus modoc* carpenter ants (n = 12–13) (**a**), and *Myrmica rubra* fire ants (n = 10) (**b**) during 21 May to 13 September 2021. Colonies were offered a choice between aqueous solutions of essential amino acids [EAAs (5.55%), w/v), sucrose (5.55%, w/v), and both [EAAs (1%), sucrose (4.55%), w/v]. Coloured symbols indicate the responses of individual colonies, and black symbols represent modelled estimated marginal means and 95% confidence intervals. Nutrient solution, date, and interaction between nutrient solution and date, were all significant predictors of bait consumption (see “Results”). Statistical results of pairwise comparisons within date are reported in Table S7.

In the field experiment with *C. modoc* colonies, bait nutrient(s), date, and interaction between bait nutrient(s) and date, were all significant predictors of bait consumption by ants (bait nutrient(s): χ^2^ = 371.1, d.f. = 18, p < 0.0001; date: χ^2^ = 117.04, d.f. = 20, p < 0.0001; interaction between bait nutrient(s) and date: χ^2^ = 109.95, d.f. = 15, p < 0.0001; Fig. 4a). Invariably over time, *C. modoc* colonies preferentially consumed EAAs, and EAAs + sucrose in a binary blend (Fig. 4a; Table S7). Consumption of water, and of sugar, decreased over time^75^ (Fig. 4a).

Similarly, in the field experiment with *M. rubra* colonies, bait nutrient(s), date, and interaction between bait nutrient(s) and date, were all significant predictors of bait consumption by ants [bait nutrient(s): χ^2^ = 403.59, d.f. = 21, p < 0.0001; date: χ^2^ = 267.51, d.f. = 24, p < 0.0001; interaction between bait nutrient(s) and date: χ^2^ = 245.17, d.f. = 18, p < 0.0001; Fig. 4b]. Across sampling dates, *M. rubra* colonies generally consumed more sucrose, and more sucrose + EAAs in a binary blend, than EAAs and water (Fig. 4b; Table S7). Sucrose consumption declined over time, whereas the consumption of sucrose in a binary blend with EAAs increased during the last three sampling dates^75^ (Fig. 4b).

### Investigating nutrient consumption of *L. niger* and *F. aserva* (Exps. 23–24; Field)

**Figure 5:**
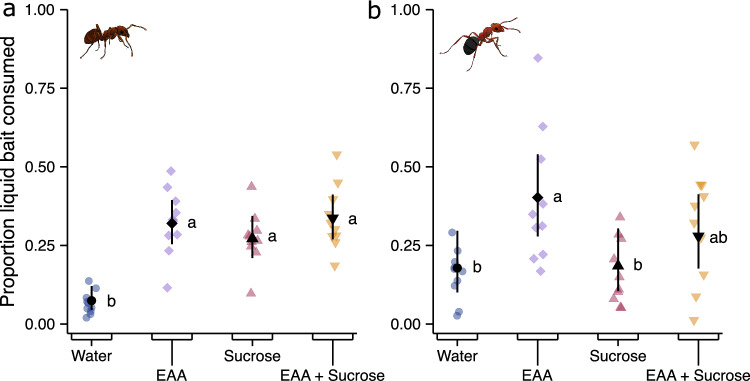
Comparative consumption of liquid food baits by colonies of *Lasius niger* black garden ants (n = 10) and *Formica aserva* thatching ants (n = 10). Colonies were offered a choice between aqueous solutions of essential amino acids [EAAs (5.55%) w/v], sucrose (5.55%, w/v), and both [EAAs (1%); sucrose (4.55%) w/v). Coloured symbols represent responses of individual colonies and black symbols are estimated marginal means and 95% confidence intervals. Treatment (bait composition) was a significant predictor of bait consumption (see results). Means with different letters are statistically different (p < 0.05) in pairwise comparisons (see Table S8).

Bait nutrients affected bait consumption by *L. niger* colonies (χ^2^ = 48.33, d.f. = 3, p < 0.0001; Fig. 5a) and *F. aserva* colonies (χ^2^ = 12.29, d.f. = 3, p = 0.006; Fig. 5b). Colonies of *L. niger* equally consumed baits containing EAAs, sucrose, and EAAs + sucrose, all of which being preferred to plain water (control stimulus) (Table S7). Colonies of *F. aserva* preferentially consumed baits containing EAAs, which they consumed more than sucrose baits but (statistically) not more than EAA + sucrose baits (Table S7).

## Discussion

Macronutrient preferences differed among the ant taxa we tested in our study which varied in size, life history, and preferred habitat. *Camponotus modoc* and *M. rubra* fed on macro-nutrients but not on micro-nutrients, and preferentially consumed specific macro-nutrients such as essential amino acids (EAAs) and sucrose. Each species, however, preferred a different macro-nutrient. *Camponotus modoc* favored nitrogenous urea and EAAs, whereas *M. rubra* favored sucrose. In contrast, neither species consumed micro-nutrients such as salts and vitamins, and lipid-related compounds such as glycerides and fatty acids. There was no shift in preferred macro-nutrient(s) over the course of the foraging season. Colonies of *C. modoc* preferentially and consistently consumed EAAs, and EEAs blended with sucrose, whereas *M. rubra* colonies generally consumed sucrose, and sucrose blended with EAAs. Macro-nutrients preferentially consumed by *C. modoc* and *M. rubra* were also readily consumed by *L. niger* and *F. aserva.* We did not observe other ant species at baits during field trials, indicating that all data were generated exclusively by our study species. Although macro-nutrient preference and consumption differed among species, the underlying drivers for these differences, such as contrasting life-history and spatiotemporal food availability, are yet to be studied.

Various species of ants distinguish between different saccharides^37–41^, and between different amino acids^49^. To date, only *Camponotus* ants have been demonstrated to consume urea (this study;^83–85^). Lipid-related compounds such as glycerides and sterols were as unappealing as water controls, and fatty acids were ingested even less than water. These results were not expected considering that ants use glycerides and fatty acids on the surface of food items as pickup cues^50–53^. Additionally, ants ingest oils^38^, although it is not known how they distinguish between oil types. The propensity of ants to salt-feed increases with distance from the ocean^54^, and is generally common in arboreal and herbivorous species^54,57^ but see^56^. However, *C. modoc* did not consume salt solutions and *M. rubra* was deterred by them*.* This may have been due, in part, to the salt composition we tested for consumption by ants. We offered a blend of salts drawing on the composition of a synthetic ant diet^74^, whereas other studies offered sodium chloride (NaCl) as a single salt. Vitamins did not elicit foraging responses in our study but B vitamin added to water improved its acceptance by the imported fire ant *Solenopsis richerti*, albeit to a lesser extent than sugars or amino acids^58^. The effects of certain vitamins on ant colony health remain inconsistent^14,86^.

Foraging preferences by ants in our study were largely driven by the specific macro-nutrient that each ant favored. Colonies of *C. modoc* and *F. aserva* preferentially foraged on the nitrogenous macro-nutrients, urea and/or EAAs, whereas *M. rubra* colonies preferentially consumed sucrose. Based on mean feeding responses, field colonies of *L. niger* consumed sucrose and EEAs equally. However, feeding preferences of individual colonies observably differed, with some colonies favoring sucrose and others EAAs^75^. These data indicate that the colonies’ preferred macro-nutrient may shift over time in accordance with the colonies’ demographics and/or resource availability or competition in their habitat. Similarly, bait selectivity by tropical ants was affected by prior feeding experience and by competition with ant community members at bait stations^37^. In contrast, field colonies of *C. modoc* and *M. rubra* consistently favored EAAs and sucrose, respectively, demonstrating persistent selection of a specific macro-nutrient. Similarly, tropical ants species preferred specific, and contrasting, blends of sucrose and particular amino acids^37^. Geometric framework studies (investigating the effects of nutrient mixtures on ant health) concluded that ants prioritise sustained carbohydrate supplies^2^ which are deemed essential for colony health^3,4,6–10^, whereas field studies revealed that many ant species prefer blends of sucrose and amino acids to sucrose alone^37^. In our season-long (21 May to 13 September) field study with *C. modoc* and *M. rubra* (Fig. 4), the blend of sucrose + EAAs was consistently consumed at a level comparable to consumption levels of EAAs or sucrose alone. Also, there was no sustained temporal shift in preferred macro-nutrient or blend of macro-nutrients, contrasting with previous reports that ants selectively seek carbohydrates or proteins at certain times during the foraging season^26–29^.

Both intrinsic and extrinsic factors affect foraging preferences in ants. As an intrinsic factor, the presence of brood motivates food and protein collections^31–33^. However, this intrinsic factor did not seem to have affected seasonal foraging patterns of *C. modoc* and *M. rubra* colonies (Fig. 4). Colonies of both species have seasonal egg production peaks, but larvae are consistently present in nests^60,87^, suggesting that fluctuations in the number of larvae may be too subtle to affect seasonal foraging activities. As an extrinsic factor, resource availability in time and space affects foraging patterns of ants, with scarce nutrients most intensely sought^34,35^. Seasonally, yellow crazy ants, *Anoplolepis gracilipes*, preferentially seek sugar-rich food in the wet season and protein-rich food in the dry season, despite brood being present in nests year round^27^. These preferences align with the shortages of protein-rich invertebrates during the dry season and sugar-rich honeydew from scale insects during the wet season^27^. Nutrient preferences may also differ according to the strata occupied by ants in an ecosystem^28,88–90^. For example, across six tropical biomes in South America, arboreal ants foraged most intensely on carbohydrates, whereas ground-nesting ants preferentially foraged on lipids^88^. Consistent preference by *C. modoc* for EAAs, and by *M. rubra* for sucrose, could imply that these resources are consistently limited and therefore are preferentially sought. That *M. rubra* equally consumed EAAs and sucrose in a laboratory experiment (Fig. 2d) but favoured sucrose, and sucrose blended with EEAs, in the season-long field experiment (Fig. 4b) could have been due to contrasting nutrients obtainable by laboratory and field colonies. In ants, both intrinsic and extrinsic factors are likely at play, simultaneously, although these interactions have not yet been explored.

Lastly, findings in our study have significant implications for control of (invasive) pest ants. Presently, leading commercial baits appeal to ‘sweet-loving ants’ but not to species that preferentially seek protein-rich food, and thus would require baits containing essential amino acids. In our study, colonies of *C. modoc* that preferentially ingested aqueous solutions of EAAs, and colonies of *M. rubra* that preferentially ingested aqueous solutions of sucrose, all consumed baits containing both EAAs and sucrose to the same extent as they consumed baits containing only their preferred macro-nutrient, indicating that the presence of a less preferred macro-nutrient as a bait constituent did not diminish the bait’s ‘appeal’. The same conclusion applies to the other ant taxa tested in this study, *L. niger* and *F. aserva*. It follows that both sucrose and EAAs could be constituents in the same bait, and thus would be appealing to both ‘sugar- and protein-loving ants’. Moreover, even if there were pest ants that shift their macro-nutrient preference over the foraging season from carbohydrates to proteins, or vice versa, both macro-nutrients would be present in the bait, thus retaining its season-long appeal to foraging ants. We favor boric acid as the lethal constituent in such a bait because—like sucrose and EAAs—it is water-soluble and once dissolved in water expresses antimicrobial activity^91,92^, thus preventing spoilage of the bait’s macro-nutrients.

### Supplementary Information

Supplementary Information.

## Data Availability

Data are available from Mendeley Data and can be accessed at: 10.17632/zbtvkyzwcj.1^75^.
