# Supplementary material for: Effects of macro- and micro-nutrients on momentary and season-long feeding responses by select species of ants
Source: Sci Rep. 2024 Mar 8;14:5727. doi: 10.1038/s41598-024-56133-y (PMC10923885; doi:10.1038/s41598-024-56133-y)
Supplement: Supplementary file 1 — Supplementary Information. [file 41598_2024_56133_MOESM1_ESM.pdf]

Figures

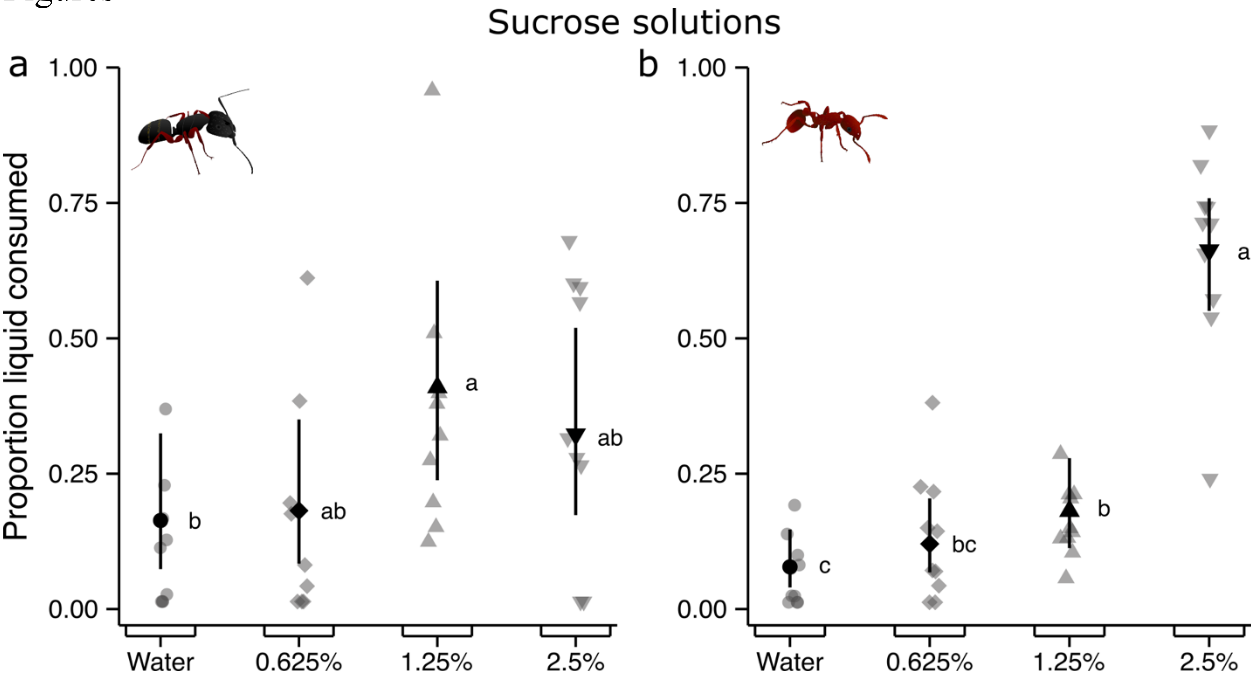

Figure S1: Proportional consumption of a plain water control and concurrently offered aqueous sucrose solutions (0.625%, 1.25% and 2.5% w/v) by colonies of *Camponotus modoc* (n = 9; **a**) and *Myrmica rubra* (n = 10; **b**). Grey symbols represent individual replicates and black symbols and whiskers denote estimated marginal means and 95% confidence intervals. Treatment (percent sugar solution) was a significant predictor of consumption for both *C. modoc* and *M. rubra* (see results). In each subpanel, different letters next to means indicate significantly different consumption levels in pairwise comparisons between means (see Table S1).

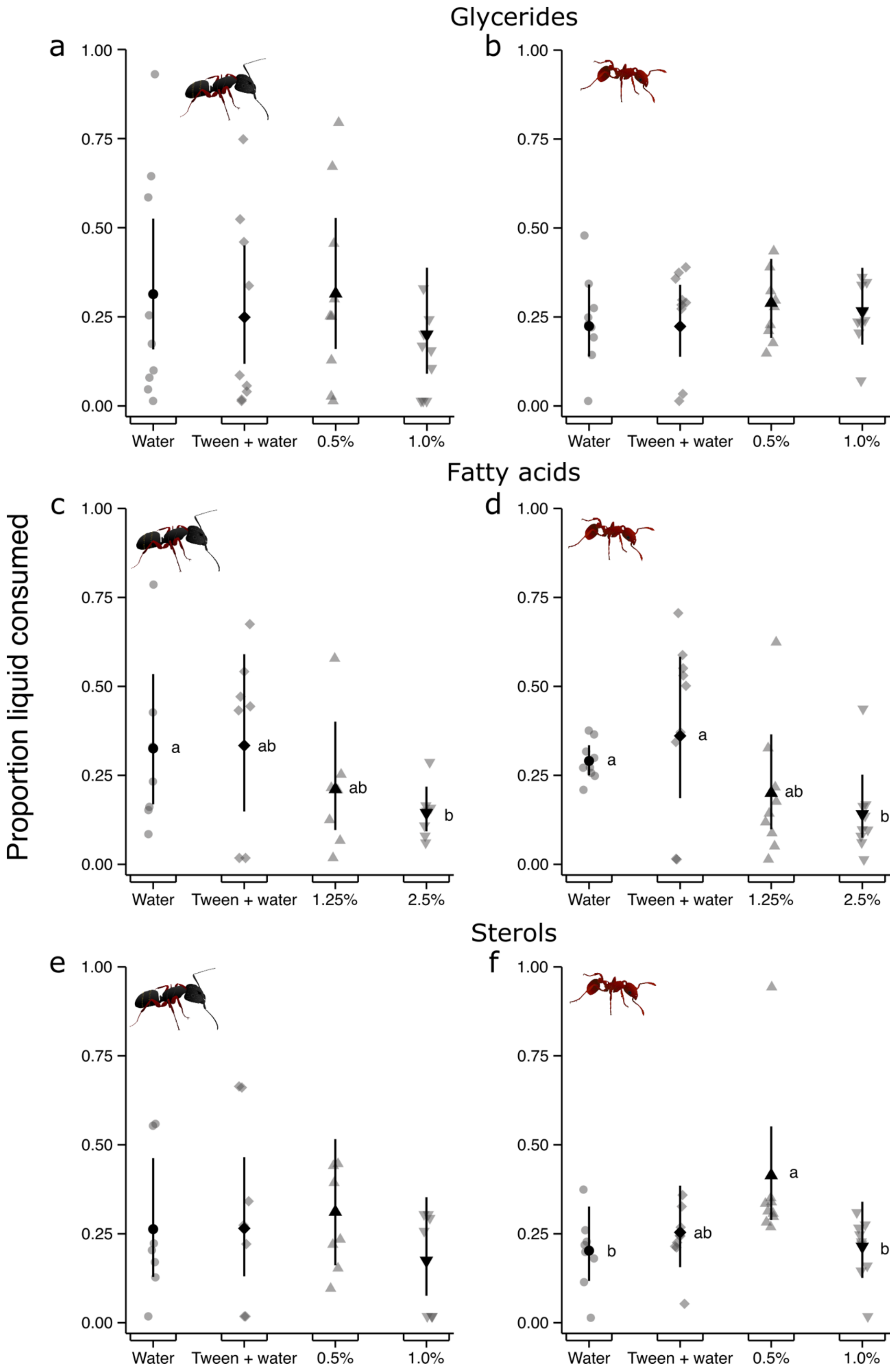

Glycerides
a
b
Fatty acids
c
d
Sterols
e
f
Proportion liquid consumed
1.00
0.75
0.50
0.25
0.00
Water
Tween + water
0.5%
1.0%
1.25%
2.5%
a
ab
b

Figure S2: Consumption of aqueous solutions of various lipids (glycerides, fatty acids, sterols) by ants. Proportional consumption by colonies of *Camponotus modoc* (**a**, n = 9; **c**, n = 7; **e**, n = 7) and *Myrmica rubra* (**b**, n = 9; **d**, n = 9, **f**, n = 9) of a water control, an aqueous Tween 80 control (0.1% w/v), and aqueous Tween 80 solutions (0.1% w/v) of (*i*) fatty acids (1.25%: 0.75% oleic acid, 0.415% linoleic, 0.085% linolenic acid; 2.5%: 1.5% oleic acid, 0.83% linoleic, 0.17% linolenic acid), (*ii*) glycerides (0.5%: 0.0625% triolein, 0.4375% diolein; 1.0%: 0.125% triolein, 0.875% diolein, and (*iii*) sterols (0.5%: 0.031% stigma sterol, 0.031% 7-dehydrocholesterol, 0.031% cholestanol, 0.44% cholesterol; 1%: 0.063% stigma sterol, 0.063% 7-dehydrocholesterol, 0.063% cholestanol, 0.81% cholesterol). Grey symbols represent individual replicates and black symbols and whiskers denote estimated marginal means and 95% confidence intervals. Treatment (type of aqueous solution) was a significant predictor of consumption for fatty acids in *C. modoc* and *M. rubra,* and for sterols in *M. rubra* (see results). In each subpanel, different letters next to means indicate significantly different consumption levels in pairwise comparisons between means (see Table S2)

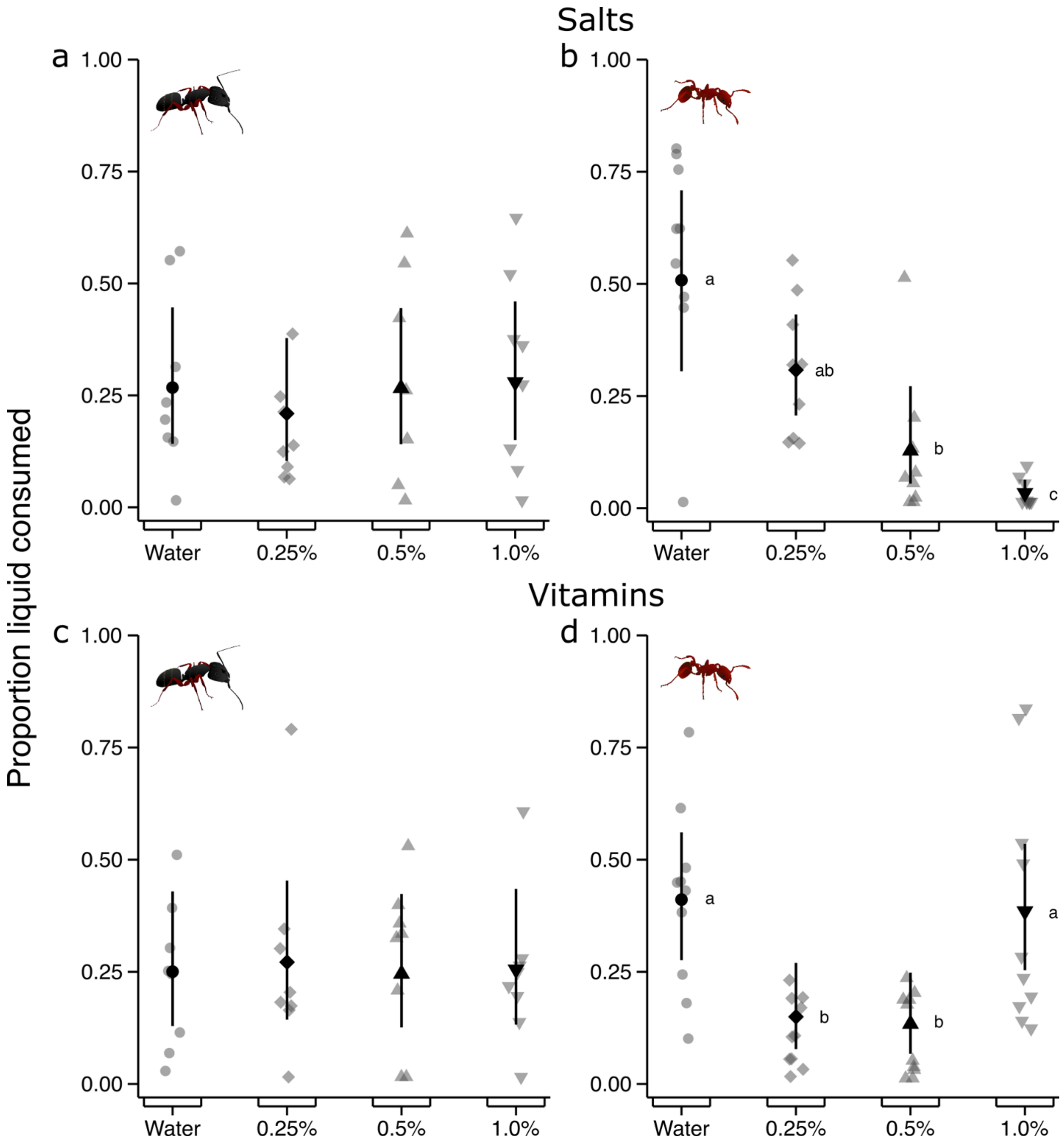

Figure S3: Consumption of aqueous salt and vitamin solutions by ants. Proportional consumption by colonies of *Camponotus modoc* (**a**, n = 8; **c**, n = 8) and *Myrmica rubra* (**b**, n = 9; **d**, n = 10) of aqueous solutions of (*i*) salts (0.25%, 0.5%, 1%; equal amounts of $CuCl_2$, $FeCl_3$, $MnCl_2$, NaCl, $ZnCl_2$, KH_2PO_4, $MgSO_4$) and (*ii*) vitamins (0.25%, 0.5%, 1%; equal amounts of *p*-amino benzoic acid, ascorbic acid, calcium pantothenate, folic acid, nicotinic acid, pyridoxin hydrochloride, riboflavin, thiamine, choline chloride). Grey symbols represent individual replicates and black symbols and whiskers denote estimated marginal means and 95%

confidence intervals. Treatment (type of aqueous solution) was a significant predictor of consumption for salts and vitamins in *M. rubra* (see results). In each subpanel, different letters next to means indicate significantly different consumption levels in pairwise comparisons between means (see Table S3).

Supplementary Tables

Table S1: Composition of nutrient test stimuli, their ratios, and purities for chemicals used in ant foraging experiments.

Test stimuli	Chemical	Fraction of total	Supplier[a]	Purity	CAS
Sucrose	D-sucrose	1.00	SA	≥99%	57-50-1
Urea	Urea	1.00	ME	≥99%	57-13-6
Essential amino acids[b]	L-Glutamic Acid	0.091	SA	99%	56-86-0
	L-Alanine	0.091	SA	≥98%	56-41-7
	L-Isoleucine	0.091	MI	≥98%	73-32-5
	L-Leucine	0.091	SA	97%	61-90-5
	L-Valine	0.091	SA	≥98%	72-18-4
	L-Tryptophan	0.091	SA	≥98%	73-22-3
	L-Arginine	0.091	SA	≥98%	74-79-3
	L-Histidine	0.091	SA	≥99%	71-00-1
	L-Threonine	0.091	SA	≥98%	72-19-5
	L-Methionine	0.091	SA	≥98%	63-68-3
	L-Phenylalanine	0.091	SA	99%	63-91-2
Glycerides[c]	Triolein	0.125	GL	NA[h]	122-32-7
	1,2-Diolein	0.875	AK	≥97%	2442-61-7
Fatty acids[d]	Oleic acid	0.600	AA	90%	112-80-1
	Linoleic acid	0.332	CB	96%	60-33-3
	Linolenic acid	0.068	CB	70%	463-40-1
Sterols[e]	stigma sterol	0.0625	SA	~95%	83-48-7
	7-dehydrocholesterol	0.0625	SA	≥95%	434-16-2
	5α-Cholestan-3β-ol	0.0625	TRC	≥95%	80-97-7
	Cholesterol	0.8125	AK	≥99%	57-88-5
Salts[f]	$CuCl_2$	0.143	SA	97%	7447-39-4
	$FeCl_3$	0.143	OW	98%	7705-08-0
	$MnCl_2$	0.143	SA	≥99%	7773-01-5
	NaCl	0.143	FI	≥99%	7647-14-5
	$ZnCl_2$	0.143	AA	≥99%	7646-85-7
	KH_2PO_4	0.143	SA	≥99%	7778-77-0
	$MgSO_4$	0.143	CL	≥99%	7487-88-9
Vitamins[g]	p-amino benzoic acid	0.111	SA	≥99%	150-13-0
	Ascorbic acid	0.111	BS	≥97%	50-81-7
	Calcium D-pantothenate	0.111	SA	≥98%	137-08-6
	Folic acid	0.111	SA	≥97%	59-30-3
	Nicotinic acid	0.111	SA	≥98%	59-67-6
	Pyridoxin hydrochloride	0.111	SA	≥98%	58-56-0
	Riboflavin	0.111	SA	≥98%	83-88-5
	Thiamine	0.111	SA	≥99%	67-03-8
	Choline chloride	0.111	SA	≥99%	67-48-1

[a] SA = Sigma Aldrich, Burlington, MA, USA; ME = Merck, Darmstadt, Germany; MI = Millipore, Burlington, MA, USA; GL = Gries-Lab synthesis (Renyard lipid); AK = AK Scientific, Inc., Union City, CA, USA; AA = Alfa Aesar, Ward Hill, MA, USA; CB = Combi-Blocks Inc., San Diego, CA, USA; TRC = Toronto Research Chemicals Inc., Toronto, ON, Canada; OW = Oakwood Products, Inc., Estill, SC, USA; FI = Fisher Scientific International, Inc., Pittsburgh, PA, USA; CL = Caledon Laboratories Ltd., Georgetown, ON, Canada

[b] Essential amino acids listed as in Feldhaar et al., 2007.

[c] The ratio of 1,2 diolein to triolein was based on limited quantity of available triolein.

[d] Fatty acids are in ratios as tested in Hughes et al., 1994.

[e] Sterols were tested in approximate dietary ratios for insects with cholesterol being the dominant component listed in Table 3, Behmer and David Nes, 2003.

[f] We used salts listed in Feldhaar et al., 2007 but tested them in equal ratio.

[g] We used vitamins listed in Feldhaar et al., 2007 but tested them in equal ratio.

[h] The purity of triolein could not be ascertained due to its high molecular weight (885) and thus poor gas chromatography.

Table S2: Modelled estimated marginal means (EMMs) and 95% confidence intervals (CIs) of proportional consumption of aqueous solutions of sucrose (S) by colonies of *Camponotus modoc* and *Myrmica rubra*. We also report pairwise comparisons between treatments and their Tukey adjusted p-values.

Exp. #	Treatment	EMMs (95% CIs)	Pairwise	Z ratio	p-value
C. modoc					
Exp. 1	Water	0.16 (0.07–0.32)	Water *vs* 0.625% S	-0.26	0.99
	0.625% S	0.18 (0.08–0.35)	Water *vs* 1.25% S	-2.66	**0.04**
	1.25% S	0.41 (0.24–0.60)	Water *vs* 2.5% S	-1.87	0.23
	2.5% S	0.32 (0.17–0.51)	0.625% S *vs* 1.25% S	-2.41	0.08
			0.625% S *vs* 2.5% S	-1.62	0.37
			1.25% S *vs* 2.5% S	0.82	0.84
M. rubra					
Exp .2	Water	0.16 (0.07–0.32)	Water *vs* 0.625% S	-1.31	0.55
	0.625% S	0.18 (0.08–0.35)	Water *vs* 1.25% S	-2.73	**0.03**
	1.25% S	0.41 (0.24–0.61)	Water *vs* 2.5% S	-9.07	**<0.0001**
	2.5% S	0.32 (0.17–0.52	0.625% *vs* 1.25% S	-1.46	0.46
			0.625% *vs* 2.5% S	-8.35	**<0.0001**
			1.25% *vs* 2.5% S	-7.4	**<0.0001**

Table S3: Modelled estimated marginal means (EMM) and 95% confidence intervals (CI) of proportional consumption of aqueous solutions of glycerides (G), fatty acids (FA), and sterols (S) by colonies of *Camponotus modoc* and *Myrmica rubra*. We also report pairwise comparisons between treatments and their Tukey adjusted p-values.

Exp. #	Treatment	EMM (95% CI)	Pairwise	Z ratio	p-value
C. modoc – Glycerides					
3	Water	0.31 (0.16–0.52)	Water *vs* Tween + water	0.65	0.91
	Tween + Water	0.25 (0.12–0.45)	Water *vs* 0.5% G	-0.02	1.0
	0.5% G	0.32 (0.16–0.53)	Water *vs* 1.0% G	1.20	0.63
	1.0% G	0.20 (0.09–0.39)	Tween + water *vs* 0.5% G	-0.66	0.91
			Tween + water *vs* 1.0% G	0.55	0.95
			0.5% G *vs* 1.0% G	1.22	0.62
M. rubra – Glycerides					
4	Water	0.22 (0.14–0.34)	Water *vs* Tween + water	0.01	1.0
	Tween + Water	0.22 (0.14–0.34)	Water *vs* 0.5% G	-1.08	0.70
	0.5% G	0.29 (0.19–0.41)	Water *vs* 1.0% G	-0.71	0.89
	1.0% G	0.27 (0.17–0.39)	Tween + water *vs* 0.5% G	-1.09	0.69
			Tween + water *vs* 1.0% G	-0.72	0.89
			0.5% G *vs* 1.0% G	0.37	0.98
C. modoc – Fatty acids					
5	Water	0.33 (0.17–0.53)	Water *vs* Tween + water	-0.07	1.0
	Tween + Water	0.33 (0.15–0.59)	Water *vs* 1.25% FA	1.17	0.64
	1.25% FA	0.21 (0.10–0.40)	Water *vs* 2.5% FA	2.61	**0.04**
	2.5% FA	0.14 (0.09–0.22)	Tween + water *vs* 1.25% FA	1.12	0.68
			Tween + water *vs* 2.5% FA	2.31	0.09
			1.25% FA *vs* 2.5% FA	1.09	0.69
M. rubra – Fatty acids					
6	Water	0.29 (0.25–0.33)	Water *vs* Tween + water	-0.86	0.83
	Tween + Water	0.36 (0.12–0.58)	Water *vs* 1.25% FA	1.4	0.48
	1.25% FA	0.20 (0.10–0.36)	Water *vs* 2.5% FA	3.05	**0.01**
	2.5% FA	0.14 (0.07–0.25)	Tween + water *vs* 1.25% FA	1.64	0.35
			Tween + water *vs* 2.5% FA	2.66	**0.04**
			1.25% FA *vs* 2.5% FA	0.96	0.77

C. modoc – Sterols					
7	Water	0.26 (0.13–0.46)	Water *vs* Tween + water	-0.02	1.0
	Tween + Water	0.27 (0.13–0.46)	Water *vs* 0.5% S	-0.49	0.96
	0.5% S	0.31 (0.16–0.52)	Water *vs* 1.0% S	1.04	0.73
	1.0% S	0.17 (0.08–0.35)	Tween + water *vs* 0.5% S	-0.47	0.97
			Tween + water *vs* 1.0% S	1.06	0.71
			0.5% S *vs* 1.0% S	1.52	0.43
M. rubra – Sterols					
8	Water	0.20 (0.12–0.33)	Water *vs* Tween + water	-0.83	0.84
	Tween + Water	0.25 (0.15–0.39)	Water *vs* 0.5% S	-3.0	**0.01**
	0.5% S	0.41 (0.29–0.55)	Water *vs* 1.0% S	-0.20	1.0
	1.0% S	0.21 (0.13–0.34)	Tween + water *vs* 0.5% S	-2.21	0.12
			Tween + water *vs* 1.0% S	0.64	0.92
			0.5% S *vs* 1.0% S	2.82	**0.02**

Table S4: Modelled estimated marginal means (EMMs) and 95% confidence intervals (CIs) of proportional consumptions of aqueous solutions of salts (S) and vitamins (V) by colonies of *Camponotus modoc* and *Myrmica rubra.* We also report pairwise comparisons between treatments and their Tukey adjusted p-values.

Exp. #	Treatment	EMMs (CIs)	Pairwise	Z ratio	p-value
C. modoc - salts					
9	Water	0.27 (0.14–0.45)	Water *vs* 0.25% S	0.71	0.89
	0.25%	0.21 (0.10–0.38)	Water *vs* 0.5% S	0.02	1.0
	0.5%	0.27 (0.14–0.45)	Water *vs* 1.0% S	-0.14	1.0
	1.0%	0.28 (0.15–0.46)	0.25% S *vs* 0.5% S	-0.70	0.90
			0.25% S *vs* 1.0% S	-0.85	0.83
			0.5% S *vs* 1.0% S	-0.15	1.0
M. rubra - salts					
10	Water	0.51 (0.31–0.71)	Water *vs* 0.25% S	2.08	0.16
	0.25%	0.31 (0.21–0.43)	Water *vs* 0.5% S	3.84	**0.0007**
	0.5%	0.13 (0.05–0.27)	Water *vs* 1.0% S	7.80	**<0.0001**
	1.0%	0.03 (0.02–0.06)	0.25% S *vs* 0.5% S	2.57	0.05
			0.25% S *vs* 1.0% S	7.45	**<0.0001**
			0.5% S *vs* 1.0% S	3.12	**0.0097**
C. modoc - vitamins					
11	Water	0.25 (0.13–0.43)	Water *vs* 0.25% V	-0.24	1.0
	0.25%	0.27 (0.14–0.45)	Water *vs* 0.5% V	0.06	1.0
	0.5%	0.25 (0.13–0.42)	Water *vs* 1.0% V	-0.06	1.0
	1.0%	0.26 (0.13–0.43)	0.25% V *vs* 0.5% V	0.30	1.0
			0.25% V *vs* 1.0% V	0.19	1.0
			0.5% V *vs* 1.0% V	-0.11	1.0
M. rubra - vitamins					
12	Water	0.41 (0.28–0.56)	Water *vs* 0.25% V	3.61	**0.002**
	0.25%	0.15 (0.08–0.27)	Water *vs* 0.5% V	3.90	**0.0006**
	0.5%	0.13 (0.07–0.25)	Water *vs* 1.0% V	0.32	0.99
	1.0%	0.39 (0.25–0.54)	0.25% V *vs* 0.5% V	0.32	0.99
			0.25% V *vs* 1.0% V	-3.33	**0.005**
			0.5% V *vs* 1.0% V	-3.62	**0.002**

Table S5: Modelled estimated marginal means (EMMs) and 95% confidence intervals (CIs) of proportional consumptions of aqueous solutions of urea, essential amino acids (EAA) or sucrose – presented singly and in ternary combination (Blend) – by colonies of *Camponotus modoc* and *Myrmica rubra*. The concentration of each macro-nutrient type was kept the same as in the ternary blend ('unadjusted') or matched the total nutrient concentration of the blend ('adjusted'). We also report pairwise comparisons between treatments and their Tukey adjusted p-values.

Exp. #	Treatment	EMMs (CI)	Pairwise	Z ratio	p-value
C. modoc – unadjusted					
13	Water	0.022 (0.015–0.033)	Water *vs* Urea	-7.73	**<0.0001**
	Urea	0.23 (0.13–0.40)	Water *vs* EAAs	-11.25	**<0.0001**
	EAAs	0.28 (0.19–0.39)	Water *vs* Sucrose	-3.14	**0.01**
	Sucrose	0.05 (0.03–0.08)	Water *vs* Blend	-20.36	**<0.0001**
	Blend	0.44 (0.39–0.50)	Urea *vs* EAAs	-0.63	0.97
			Urea *vs* Sucrose	5.02	**<0.0001**
			Urea *vs* Blend	-3.13	**0.01**
			EAAs *vs* Sucrose	7.22	**<0.0001**
			EAAs *vs* Blend	-3.46	**0.005**
			Sucrose *vs* Blend	-12.82	**<0.0001**
C. modoc – adjusted					
14	Water	0.03 (0.02–0.04)	Water *vs* Urea	-6.59	**<0.0001**
	Urea	0.19 (0.10–0.31)	Water *vs* EAAs	-11.34	**<0.0001**
	EAAs	0.44 (0.30–0.59)	Water *vs* Sucrose	-2.83	**0.04**
	Sucrose	0.06 (0.03–0.10)	Water *vs* Blend	-8.50	**<0.0001**
	Blend	0.32 (0.19–0.50)	Urea *vs* EAAs	-3.49	**0.004**
			Urea *vs* Sucrose	3.75	**0.002**
			Urea *vs* Blend	-1.89	0.32
			EAAs *vs* Sucrose	7.83	**<0.0001**
			EAAs *vs* Blend	1.37	0.65
			Sucrose *vs* Blend	-5.65	**<0.0001**
M. rubra – unadjusted					
15	Water	0.07 (0.04–0.12)	Water *vs* Urea	-0.41	0.99
	Urea	0.08 (0.05–0.13)	Water *vs* EAAs	-3.36	**0.007**
	EAAs	0.16 (0.11–0.22)	Water *vs* Sucrose	-7.09	**<0.0001**
	Sucrose	0.32 (0.25–0.40)	Water *vs* Blend	-8.07	**<0.0001**
	Blend	0.38 (0.30–0.45)	Urea *vs* EAAs	-2.98	**0.02**
			Urea *vs* Sucrose	-6.81	**<0.0001**

			Urea *vs* Blend	-7.82	**<0.0001**
			EAAs *vs* Sucrose	-4.31	**0.0002**
			EAAs *vs* Blend	-5.50	**<0.0001**
			Sucrose *vs* Blend	-1.29	0.70
M. rubra – adjusted					
16	Water	0.06 (0.04–0.10)	Water *vs* Urea	1.52	0.55
	Urea	0.04 (0.03–0.07)	Water *vs* EAAs	-11.73	**<0.0001**
	EAAs	0.39 (0.33–0.44)	Water *vs* Sucrose	-9.98	**<0.0001**
	Sucrose	0.31 (0.27–0.37)	Water *vs* Blend	-7.41	**<0.0001**
	Blend	0.23 (0.18–0.27)	Urea *vs* EAAs	-12.19	**<0.0001**
			Urea *vs* Sucrose	-10.64	**<0.0001**
			Urea *vs* Blend	-8.37	**<0.0001**
			EAAs *vs* Sucrose	2.47	0.09
			EAAs *vs* Blend	5.73	**<0.0001**
			Sucrose *vs* Blend	3.32	**0.008**

Table S6: Modelled estimated marginal means (EMMs) and 95% confidence intervals (CIs) of proportional consumptions of aqueous solutions of urea, essential amino acids (EAAs) or sucrose – presented in binary combinations and in a ternary blend (Blend) – by colonies of *Camponotus modoc* and *Myrmica rubra*. The concentrations and ratios of macro-nutrients in binary combinations were kept the same as in the ternary blend ('unadjusted'), or matched – at equivalent proportion – the total concentration of the ternary blend ('adjusted'). We also report pairwise comparisons between treatments and their Tukey adjusted p-values.

Exp. #	Treatment	EMMs (CIs)	Pairwise	Z ratio	p-value
C. modoc - unadjusted					
17	Water	0.03 (0.02–0.05)	Water *vs* Urea + EAAs	-8.91	**<0.0001**
	Urea + EAAs	0.33 (0.21–0.46)	Water *vs* Urea + Sucrose	-4.48	**<0.0001**
	Urea + Sucrose	0.16 (0.07–0.32)	Water *vs* EAAs + Sucrose	-6.57	**<0.0001**
	EAA + Sucrose	0.23 (0.13–0.37)	Water *vs* Blend	-11.41	**<0.0001**
	Blend	0.29 (0.25–0.34)	Urea + EAAs *vs* Urea + Sucrose	2.25	0.16
			Urea + EAAs *vs* EAAs + Sucrose	1.38	0.64
			Urea + EAAs *vs* Blend	0.62	0.97
			Urea + Sucrose *vs* EAAs + Sucrose	-1.01	0.85
			Urea + Sucrose *vs* Blend	-2.18	0.19
			EAAs + Sucrose *vs* Blend	-1.17	0.77
C. modoc - adjusted					
18	Water	0.03 (0.01–0.05)	Water *vs* Urea + EAAs	-8.33	**<0.0001**
	Urea + EAAs	0.39 (0.25–0.55)	Water *vs* Urea + Sucrose	-4.78	**<0.0001**
	Urea + Sucrose	0.15 (0.08–0.25)	Water *vs* EAAs + Sucrose	-5.90	**<0.0001**
	EAA + Sucrose	0.21 (0.12–0.35)	Water *vs* Blend	-8.41	**<0.0001**
	Blend	0.26 (0.20–0.34)	Urea + EAA *vs* Urea + Sucrose	3.52	**0.004**
			Urea + EAAs *vs* EAAs + Sucrose	2.28	0.15
			Urea + EAAs *vs* Blend	1.97	0.28
			Urea + Sucrose *vs* EAAs + Sucrose	-1.18	0.76
			Urea + Sucrose *vs* Blend	-2.42	0.11
			EAA + Sucrose *vs* Blend	-0.90	0.90
M. rubra - unadjusted					
19	Water	0.09 (0.06–0.13)	Water *vs* Urea + EAAs	-0.69	0.96
	Urea + EAAs	0.11 (0.07–0.15)	Water *vs* Urea + Sucrose	-3.77	**0.002**
	Urea + Sucrose	0.18 (0.14–0.23)	Water *vs* EAAs + Sucrose	-8.34	**<0.0001**
	EAA + Sucrose	0.33 (0.27–0.39)	Water *vs* Blend	-7.40	**<0.0001**
	Blend	0.29 (0.24–0.35)	Urea + EAAs *vs* Urea + Sucrose	-3.11	**0.02**

			Urea + EAAs *vs* EAAs + Sucrose	-7.84	**<0.0001**
			Urea + EAAs *vs* Blend	-6.85	**<0.0001**
			Urea + Sucrose *vs* EAAs + Sucrose	-5.13	**<0.0001**
			Urea + Sucrose *vs* Blend	-4.03	**0.0005**
			EAA + Sucrose *vs* Blend	1.14	0.78
M. rubra - adjusted					
20	Water	0.05 (0.04–0.07)	Water *vs* Urea + EAAs	-6.79	**<0.0001**
	Urea + EAAs	0.14 (0.12–0.17)	Water *vs* Urea + Sucrose	-6.42	**<0.0001**
	Urea + Sucrose	0.14 (0.11–0.17)	Water *vs* EAAs + Sucrose	-17.74	**<0.0001**
	EAAs + Sucrose	0.45 (0.41–0.49)	Water *vs* Blend	-11.62	**<0.0001**
	Blend	0.25 (0.22–0.29)	Urea + EAAs *vs* Urea + Sucrose	0.43	0.99
			Urea + EAAs *vs* EAA + Sucrose	-14.38	**<0.0001**
			Urea + EAAs *vs* Blend	-6.03	**<0.0001**
			Urea + Sucrose *vs* EAAs + Sucrose	-14.69	**<0.0001**
			Urea + Sucrose *vs* Blend	-6.44	**<0.0001**
			EAA + Sucrose *vs* Blend	9.14	**<0.0001**

Table S7: Modelled estimated marginal means (EMMs) and 95% confidence intervals (CIs) of proportional consumptions of aqueous solutions of essential amino acids (EAAs), sucrose (S), or both (S + EAAs) by field colonies of *Camponotus modoc* and *Myrmica rubra.* We compared proportional consumption of nutrient treatments on each of 6 dates (*C. modoc*) and 7 dates (*M. rubra*). We also report pairwise comparisons between treatments within a given date and their Tukey adjusted p-values. For each date, different letters in parentheses behind treatments indicate statistically significant differences in consumptions of nutrient solutions.

Exp. #	2021	Treatment	EMMs (CIs)	Pairwise	Z ratio	p-value
C. modoc						
21	18 June	Water (b)	0.12 (0.05–0.24)	Water *vs* EAAs	-4.25	**0.0001**
		EAAs (a)	0.43 (0.31–0.55)	Water *vs* Sucrose	0.58	0.94
		Sucrose (b)	0.10 (0.05–0.17)	Water *vs* S + EAAs	-3.59	**0.002**
		S + EAAs (a)	0.37 (0.25–0.50)	EAA *vs* Sucrose	5.94	**<0.0001**
				EAA *vs* S + EAAs	0.81	0.85
				Sucrose *vs* S + EAAs	-5.08	**<0.0001**
	03 July	Water (c)	0.05 (0.03–0.10)	Water *vs* EAAs	-7.25	**<0.0001**
		EAAs (a)	0.42 (0.30–0.55)	Water *vs* Sucrose	-2.92	**0.02**
		Sucrose (b)	0.16 (0.08–0.28)	Water *vs* S + EAAs	-7.20	**<0.0001**
		S + EAAs (a)	0.38 (0.28–0.49)	EAA *vs* Sucrose	3.65	**0.002**
				EAA *vs* S + EAAs	0.57	0.94
				Sucrose *vs* S + EAAs	-3.40	**0.004**
	23 July	Water (b)	0.09 (0.05–0.15)	Water *vs* EAAs	-5.15	**<0.0001**
		EAAs (a)	0.41 (0.25–0.58)	Water *vs* Sucrose	-0.61	0.93
		Sucrose (b)	0.11 (0.06–0.20)	Water *vs* S + EAAs	-5.84	**<0.0001**
		S + EAAs (a)	0.35 (0.26–0.45)	EAA *vs* Sucrose	4.19	**0.0002**
				EAA *vs* S + EAAs	0.72	0.89
				Sucrose *vs* S + EAAs	-4.44	**0.0001**
	09 Aug.	Water (c)	0.012 (0.092–0.016)	Water *vs* EAAs	-21.54	**<0.0001**
		EAAs (a)	0.58 (0.46–0.69)	Water *vs* Sucrose	-1.78	0.28
		Sucrose (c)	0.016 (0.012–0.021)	Water *vs* S + EAAs	-15.95	**<0.0001**
		S + EAAs (b)	0.39 (0.27–0.52)	EAA *vs* Sucrose	20.26	**<0.0001**
				EAA *vs* S + EAAs	2.65	**0.04**
				Sucrose *vs* S + EAAs	-14.81	**<0.0001**

	24 Aug.	Water (b)	0.05 (0.03–0.09)	Water *vs* EAAs	-9.31	**<0.0001**
		EAAs (a)	0.44 (0.35–0.55)	Water *vs* Sucrose	-1.01	0.75
		Sucrose (b)	0.08 (0.04–0.14)	Water *vs* S + EAAs	-9.72	**<0.0001**
		S + EAAs (a)	0.43 (0.35–0.51)	EAA *vs* Sucrose	7.26	**<0.0001**
				EAA *vs* S + EAAs	0.32	0.99
				Sucrose *vs* S + EAAs	-7.45	**<0.0001**
	07 Sept.	Water (c)	0.04 (0.03–0.06)	Water *vs* EAAs	-14.76	**<0.0001**
		EAAs (a)	0.60 (0.48–0.70)	Water *vs* Sucrose	-0.60	0.93
		Sucrose (c)	0.05 (0.03–0.07)	Water *vs* S + EAAs	-7.08	**<0.0001**
		S + EAAs (b)	0.30 (0.17–0.47)	EAA *vs* Sucrose	13.65	**<0.0001**
				EAA *vs* S + EAAs	3.62	**0.002**
				Sucrose *vs* S + EAAs	-6.52	**<0.0001**
M. rubra						
22	21 May	Water (c)	0.004 (0.002–0.007)	Water *vs* EAAs	-12.98	**<0.0001**
		EAAs (b)	0.10 (0.07–0.14)	Water *vs* Sucrose	-20.75	**<0.0001**
		Sucrose (a)	0.45 (0.36–0.54)	Water *vs* S + EAAs	-22.34	**<0.0001**
		S + EAAs (a)	0.45 (0.38–0.52)	EAA *vs* Sucrose	-9.46	**<0.0001**
				EAA *vs* S + EAAs	-10.62	**<0.0001**
				Sucrose *vs* S + EAAs	-0.08	1.0.
	17 June	Water (d)	0.003 (0.002–0.004)	Water *vs* EAAs	-20.54	**<0.0001**
		EAA (c)	0.19 (0.15–0.25)	Water *vs* Sucrose	-24.57	**<0.0001**
		Sucrose (b)	0.36 (0.29–0.42)	Water *vs* S + EAAs	-28.89	**<0.0001**
		S + EAAs (a)	0.45 (0.40–0.50)	EAA *vs* Sucrose	-4.87	**<0.0001**
				EAA *vs* S + EAAs	-8.45	**<0.0001**
				Sucrose *vs* S + EAAs	-2.83	**0.02**
	04 July	Water (c)	0.03 (0.02–0.04)	Water *vs* EAAs	-10.63	**<0.0001**
		EAAs (b)	0.20 (0.15–0.26)	Water *vs* Sucrose	-16.13	**<0.0001**
		Sucrose (a)	0.40 (0.34–0.47)	Water *vs* S + EAAs	-13.08	**<0.0001**
		S + EAAs (a)	0.37 (0.28–0.48)	EAA *vs* Sucrose	-5.67	**<0.0001**
				EAA *vs* S + EAAs	-4.02	**0.0003**
				Sucrose *vs* S + EAAs	0.52	0.95

25 July	Water (b)	0.23 (0.20–0.26)	Water *vs* EAAs	-0.30	0.99
	EAAs (b)	0.24 (0.21–0.26)	Water *vs* Sucrose	-0.98	0.76
	Sucrose (b)	0.25 (0.23–0.26)	Water *vs* S + EAAs	-3.83	**0.0007**
	S + EAAs (a)	0.29 (0.27–0.31)	EAA *vs* Sucrose	-0.79	0.86
			EAA *vs* S + EAAs	-4.27	**0.0001**
			Sucrose *vs* S + EAAs	-3.98	**0.0004**
11 Aug.	Water (c)	0.01 (0.007–0.016)	Water *vs* EAAs	-14.13	**<0.0001**
	EAAs (b)	0.26 (0.18–0.36)	Water *vs* Sucrose	-15.57	**<0.0001**
	Sucrose (ba)	0.31 (0.23–0.41)	Water *vs* S + EAAs	-20.65	**<0.0001**
	S + EAAs (a)	0.42 (0.35–0.49)	EAA *vs* Sucrose	-0.89	0.81
			EAA *vs* S + EAAs	-3.18	**0.008**
			Sucrose *vs* S + EAAs	-2.25	0.11
01 Sept.	Water (d)	0.003 (0.002–0.005)	Water *vs* EAAs	-19.03	**<0.0001**
	EAAs (c)	0.19 (0.16–0.24)	Water *vs* Sucrose	-19.85	**<0.0001**
	Sucrose (b)	0.32 (0.25–0.40)	Water *vs* S + EAAs	-24.08	**<0.0001**
	S + EAAs (a)	0.48 (0.42–0.55)	EAA *vs* Sucrose	-3.61	**0.002**
			EAA *vs* S + EAAs	-9.00	**<0.0001**
			Sucrose *vs* S + EAAs	-3.91	**0.0005**
13 Sept.	Water (c)	0.003 (0.002–0.006)	Water *vs* EAAs	-10.76	**<0.0001**
	EAAs (b)	0.16 (0.08–0.28)	Water *vs* Sucrose	-11.22	**<0.0001**
	Sucrose (b)	0.22 (0.11–0.39)	Water *vs* S + EAAs	-19.58	**<0.0001**
	S + EAAs (a)	0.60 (0.47–0.71)	EAA *vs* Sucrose	-0.96	0.77
			EAA *vs* S + EAAs	-5.80	**<0.0001**
			Sucrose *vs* S + EAAs	-4.34	**0.0001**

Table S8: Modelled estimated marginal means (EMMs) and 95% confidence intervals (CIs) of proportional consumptions of aqueous solutions of essential amino acids (EAA), sucrose (S), or both (S + EAAs) by field colonies of *Lasius niger* and *Formica aserva*. We also report pairwise comparisons between treatments and their Tukey adjusted p-values.

Exp. #	Treatment	EMMs (CIs)	Pairwise	Z ratio	p-value
L. niger					
23	Water	0.07 (0.04–0.12)	Water *vs* EAAs	-7.07	**<0.0001**
	EAAs	0.32 (0.25–0.39)	Water *vs* Sucrose	-6.08	**<0.0001**
	Sucrose	0.27 (0.21–0.34)	Water *vs* S + EAAs	-7.39	**<0.0001**
	S + EAAs	0.34 (0.27–0.41)	EAAs *vs* Sucrose	1.24	0.60
			EAAs *vs* S + EAAs	-0.42	0.98
			Sucrose *vs* S + EAAs	-1.65	0.35
F. aserva					
24	Water	0.18 (0.10–0.30)	Water *vs* EAAs	-3.28	**0.006**
	EAAs	0.40 (0.28–0.54)	Water *vs* Sucrose	-0.11	1.0
	Sucrose	0.18 (0.10–0.30)	Water *vs* S + EAAs	-1.65	0.35
	S + EAAs	0.28 (0.18–0.41)	EAAs *vs* Sucrose	3.17	**0.008**
			EAAs *vs* S + EAAs	1.69	0.33
			Sucrose *vs* S + EAAs	-1.54	0.41
